# Ecotoxicological Properties of Titanium Dioxide Nanomorphologies in *Daphnia magna*

**DOI:** 10.3390/nano13050927

**Published:** 2023-03-03

**Authors:** Freddy Mendoza-Villa, Noemi-Raquel Checca-Huaman, Juan A. Ramos-Guivar

**Affiliations:** 1 Grupo de Investigación de Nanotecnología Aplicada para Biorremediación Ambiental, Energía, Biomedicina y Agricultura (NANOTECH), Facultad de Ciencias Físicas, Universidad Nacional Mayor de San Marcos, Av. Venezuela Cdra 34 S/N, Ciudad Universitaria, Lima 15081, Peru; 2 Centro Brasileiro de Pesquisas Físicas (CBPF), R. Xavier Sigaud, 150, Urca, Rio de Janeiro 22290-180, Brazil

**Keywords:** aquatic biota, metal-oxides, *biomarkers*, nanoparticles, nanowires

## Abstract

In this work, the structural, vibrational, morphological, and colloidal properties of commercial 15.1 nm TiO_2_ nanoparticles (NPs) and nanowires (NWs, 5.6 thickness, 74.6 nm length) were studied with the purpose of determining their ecotoxicological properties. This was achieved by evaluating acute ecotoxicity experiments carried out in the environmental bioindicator *Daphnia magna*, where their 24-h lethal concentration (*LC_50_*) and morphological changes were evaluated using a TiO_2_ suspension (pH = 7) with point of zero charge at 6.5 for TiO_2_ NPs (hydrodynamic diameter of 130 nm) and 5.3 for TiO_2_ NWs (hydrodynamic diameter of 118 nm). Their *LC_50_* values were 157 and 166 mg L^−1^ for TiO_2_ NWs and TiO_2_ NPs, respectively. The reproduction rate of *D. magna* after fifteen days of exposure to TiO_2_ nanomorphologies was delayed (0 pups for TiO_2_ NWs and 45 neonates for TiO_2_ NPs) in comparison with the negative control (104 pups). From the morphological experiments, we may conclude that the harmful effects of TiO_2_ NWs are more severe than those of 100% anatase TiO_2_ NPs, likely associated with brookite (36.5 wt. %) and protonic trititanate (63.5 wt. %) presented in TiO_2_ NWs according to Rietveld quantitative phase analysis. Specifically, significant change in the heart morphological parameter was observed. In addition, the structural and morphological properties of TiO_2_ nanomorphologies were investigated using X-ray diffraction and electron microscopy techniques to confirm the physicochemical properties after the ecotoxicological experiments. The results reveal that no alteration in the chemical structure, size (16.5 nm for TiO2 NPs and 6.6 thickness and 79.2 nm length for NWs), and composition occurred. Hence, both TiO_2_ samples can be stored and reused for future environmental purposes, e.g., water nanoremediation.

## 1. Introduction

Promising metal/metal-oxide nanomaterials have been synthesized by different chemical, physical, and biological synthesis techniques [1,2], such as pulse laser ablation, high-energy ball milling, sputtering, chemical reduction, microemulsion, sol gel, and green synthesis, to create different shapes of nanoparticles (NPs) [1,2]. Therefore, different enhanced physicochemical properties are expected and will differ in the required applications/purposes [2]. One of these applications is to solve existing environmental problems such as the accelerated consumption of fossil fuels through the removal of emerging pollutants that will end up in aquatic ecosystems, etc. [1,3,4]. One important nanomaterial is titanium dioxide (TiO_2_), the world production of which is estimated to reach 2.5 million tons in 2025 [5]. These are found in applications such as sun creams and self-cleaning products due to their photocatalytic, antimicrobial, and UV protection properties [6,7]. Hence, due to the high production of these nanomaterials at industrial levels, it is expected that they will be spread to aqueous bodies and represent a toxic material to water specimens as well. The ultimate process for these nanomaterials in aquatic ecosystems is a current concern and previous toxic effect evaluations have been reported [8,9,10].

Moreover, though TiO_2_ types have shown high removal efficiency in the uptake of heavy metals from water bodies [11], studies on their removal efficiency are limited to preliminary studies performed in nanoremediation. A second and mandatory study is to evaluate their ecotoxicological risks. This represents an open gap in the field of nanoremediation, since NPs will have different toxic effects depending on their size, morphologies, and textural properties (porosity +specific surface area). *Daphnia magna* (*D. magna*) is a crustacean of the cladoceran family found in lakes, ponds, etc. [12] and is an important environmental bioindicator, due to its easy handling, rapid growth, and asexual reproduction (parthenogenesis) under favorable conditions, characteristics that allowing its observation because of its biological sensitivity and response to the toxic effects of nanomaterials under standardized laboratory conditions, it is thus highly advantageous as a test specimen for ecotoxicological evaluation, e.g., *LC_50_* determination [8].

In the last years, innovative, and novel research about ecotoxicological properties (lab conditions) of metal oxides and magnetic oxides has been developed [13,14,15]. For example, Liu et al. [13] studied the chronic toxicity of the crystalline forms of TiO_2_ (rutile + anatase) NPs on the physiological parameters of *D. magna*. They found a direct relation between the energy gaps of the TiO_2_ forms (0.25 to 1 mg L^−1^) and the toxicity in the aquatic organisms. Nevertheless, these concentrations are smaller than others used for water treatments [16] and the 48h-*EC_50_* values were the same for the five samples, having a value of 100 mg L^−1^. In another study, Novak et al. [14] implemented and improved the commercial EN ISO 6341:2014 in order to analyze the effect of TiO_2_ NPs in *Daphnia magna* for 48 h postexposure experiments. No remarkable toxic effects were observed during the tested 48 h, but the *daphnids* immobility increased when testing longer exposure times, up to 72 h. This problem was solved by transferring the *daphnids* to clean water after 48 h tests. Nevertheless, no characterization of the TiO_2_ NPs was carried out to evaluate their reuse and storage. Furthermore, various pilot experiments using TiO_2_ NPs have been developed to treat polluted water using solar assistant [17] and photolitically produced hydrogen has also become a topic of research [18]. However, the handling, reuse, storage and, more importantly, the ecotoxicological properties of TiO_2_ NPs have not been studied, pilot experiments are therefore required for larger amounts of material.

Hence, determining whether concentrations of TiO_2_ (diverse nanomorphologies) are harmful to aquatic organisms is a priority in order to analyze its ecotoxicological behavior before its commercialization as a potential nanoremediator candidate. These novel results may also be used to help future markets interested in nanoremediation to establish political guidelines. More importantly, research on the ecotoxicological effects of TiO_2_ (different morphologies) on environmental biomarkers such as *D. magna* is scarce in many countries that face pollution problems [9]. Therefore, nanotoxicology experiments should be evaluated before adsorption experiments to determine lethal concentrations (*LC_50_*). More importantly, the colloidal parameters and reusability properties of TiO_2_ NPs have not been widely studied in the literature when studying the ecotoxicological properties, and the dispersion preparation method, the hydrodynamic diameter, zeta potential, and recovery properties can affect the *LC_50_* determination [8,9,10,11]. Most reports only showed ecotoxicological evaluation and this can represent a disadvantage since the reusability or conserved properties of TiO_2_ NPs were not proved.

Thus, the present work seeks to elucidate the toxic effects of TiO_2_ on *D. magna* under laboratory conditions that simulate their corresponding habitat, and then compare the results with the literature and discuss whether they are useful for the simultaneous nanoremediation of water bodies. For this, the environmental bioindicator *D. magna* was used to perform the ecotoxicity experiments for various TiO_2_ NPs and TiO_2_ nanowires (NWs) concentrations. Before studying the ecotoxicological properties of both materials, their structural, vibrational, morphological, and colloidal properties were investigated. Then, a 24 h-*LC_50_* estimation was undertaken for both commercial nanosystems using non-linear approaches. Morphological analysis was undertaken to analyze the significant damage caused to *D. magna* individuals after exposure to the material. Novel post-ecotoxicological experiments regarding the structural and morphological characterization of the recovered nanosystems were undertaken by X-ray diffraction (XRD), selected area electron diffraction (SAED), and electron energy loss spectroscopy (EELS) to ensure their reusability and regeneration properties.

## 2. Materials and Methods

### 2.1. Chemicals

To perform the ecotoxicological experiments in the presence of the environmental bioindicator *D. magna*, both 20 nm anatase TiO_2_ NPs and TiO_2_ NWs (10 µm in length and 10 nm in thickness) were purchased from SIGMA-ALDRICH without any other chemical modifications. Both samples were synthesized by the hydrothermal method.

### 2.2. Characterization of the TiO_2_ Samples

X-ray diffraction data were obtained using an Empyrean diffractometer operating with CuK_α_, 45 kV and 40 mA. X-ray diffractograms were collected in step scanning mode, 2θ = 10–100°, step of 0.026°, and 20 s/step. By means of the Match v3 software [19], the phase identification and indexation of both samples was carried out, resulting in a monophasic sample (anatase with a crystallographic chart #500-0024) for the NPs and a biphasic sample (brookite with a crystallographic chart #900-9088 and protonic trititanate with a crystallographic chart crystallography #433-6946) for the NWs. The Rietveld refinement was applied from the crystallographic information file (cif) of both samples using the FullProf Suite software (Gif sur Yvette Cedex, France, version January 2021). To refine the X-ray diffraction profile, the Thompson–Cox–Hastings (TCH) pseudo-Voigt axial divergence asymmetry function was used. The instrumental resolution function (IRF) of the diffractometer was obtained from an aluminum-oxide (Al_2_O_3_) standard with refined Caglioti parameters, U = 0.0093, V = −0.0051, and W = 0.0013 [20,21].

The μ-Raman spectrum was measured in a Renishaw inVia Raman microscope. A wavelength of λ = 785 nm (initial laser power of 82.8 mW) was used as the excitation source. An optical objective of 50× magnification was chosen for the measurements. The Raman mode profiles (spectrum taken at laser power fraction of 10% over the sample) were fit using Lorentzian functions.

The colloidal properties by means of effective hydrodynamic diameter and Zeta potential of the TiO_2_ suspensions (both morphologies) were determined using a Brookhaven Nanobrook 90 Plus PALS. The zeta potential of the suspensions as a function of pH was obtained for TiO_2_ samples to find the point of zero charge. In addition, the hydrodynamic diameter at different pH values (2, 5, 7, 9, 12) were measured for initial 50 mg $L^{-1}$ TiO_2_ NPs and NWs. All the experiments were done in duplicate. The base line index criterium was used to analyze the correct measurement.

To determine the average particle size, particle size distribution, and morphological features, the 200 kV JEOL 2100F (Tokyo, Japan) imaging electron microscopy equipment was employed in transmission (TEM), scanning (STEM), and high-resolution modes. For particle size distribution (PSD) estimation, a total number of particles and wires of 800 to 1000 was counted from 30–35 images using the Image J software. A log-normal distribution was considered to fit the obtained histograms according to [22]. Finally, the polydispersity values were estimated from the standard deviation of the log-normal distribution. The microscope has two accessories for energy-dispersive X-ray spectroscopy (EDS) and electron energy loss spectroscopy (EELS) (EELS-GIF Tridiem GATAN). Both techniques were used to evaluate the atomic composition at the nanoscale using the mode STEM. EELS experiments were carried out in the STEM imaging mode using a spot size of 0.7 nm. The spectrometer aperture was 5 mm and the energy resolution (measured by the FWHM of the zero-loss peaks) was approximately 1.8 eV. For the morphological analysis, commercial samples without any modification were labeled as before samples and recovered samples after ecotoxicological experiments were labeled as after. This nomenclature was also used ahead in this work.

### 2.3. D. magna Culture

The set of *D. magna* individuals used for the ecotoxicity experiments were cultivated in the laboratory under optimized conditions which resemble their habitat. *D. magna* were maintained in an 8:16 h light:dark photoperiod and a temperature of (20 ± 1) °C and pH = 7.5 ± 0.5. The required hatchlings were obtained by separating 120 adult *daphnia* individuals with the potential to give hatchlings in a short time. The *daphnia* individuals were fed daily with microalgae of the genus Scenedesmus, in relation to the volume of the beaker, with a scale of 1 mL per 100 mL [12].

These *D. magna* individuals were separated in to four beakers with 200 mL of standardized water for the cultivation of *D. magna*, after one to five days the corresponding neonates were born, which are clones due to the favorable conditions in which they were found. Neonates are *D. magna* individuals with a life span of less than 24 h. If *daphnia* are well fed, they can have up to 65 hatchlings in a single litter, with an average size of 12 hatchlings per litter [23].

When the 120 *D. magna* individuals were separated in the four reproduction beakers no more hatchlings were generated. Then, they were returned to the original culture beaker and other *D. magna* with the potential to give hatchlings in a short time were separated again with the purpose of continuing the ecotoxicity experiment.

It should also be highlighted that the set of *D. magna* must have a specific culture temperature, since being a bioindicator, it is very sensitive to sudden physicochemical changes that occur in its habitat. For example, a sudden change in temperature inside the beaker during feeding, oxygen concentration, etc. [23], under the specific laboratory conditions.

### 2.4. D. magna Exposure Protocol

The TiO_2_ concentrations for the ecotoxicity experiment were done separately. In a volume of 200 mL of standardized water, five concentrations of TiO_2_ NPs were prepared: 37.5 mg $L^{-1}$, 75 mg $L^{-1}$, 150 mg $L^{-1}$, 300 mg $L^{-1}$, and 600 mg $L^{-1}$. While for the TiO_2_ NWs five chosen concentrations were also prepared: 50 mg $L^{-1}$, 100 mg $L^{-1}$, 200 mg $L^{-1}$, 400 mg $L^{-1}$, and 800 mg $L^{-1}$. The individuals for each concentration were 10 neonates of *D. magna*. Experiments were done in duplicate and the mean values were reported.

### 2.5. Morphological Analysis of D. magna

The morphological evaluation was undertaken after 16 days, one day for the exposure with the TiO_2_ samples. After the 24-h exposure, surviving *D. magna* were moved to another beaker with conditions prior to TiO_2_ exposure. The remaining 15 days were for the control of the surviving *D. magna*. On the last day of the experiment, the length (micrometers) of the tail, body, antenna, eye, and heart morphological parameters of the surviving *D. magna* were measured using an optical microscope and ToupView software on a computer.

Within two weeks, *D. magna* reaches sexual maturity (generally between the sixth and ninth day [23]) to be able to reproduce either sexually or asexually depending on habitat conditions. In the ecotoxicity experiment it was evidenced that the negative control *D. magna* began to have newborns from the ninth day.

Finally, the statistical analysis of the morphological parameters was analyzed by means of the paired Student’s *t*-test using the *p* value criterium of 0.05 for the corresponding significance. A box plot was used to visualize the comparison of morphological parameters between exposed *daphnia* and negative control *D. magna*. These calculations were made using SPSS 27 statistical software. The comparison was made with respect to the negative control, a set of *D. magna* that were not exposed to the TiO_2_ sample.

## 3. Results and Discussions

### 3.1. Rietveld Refinement Analysis

**Figure 1:**
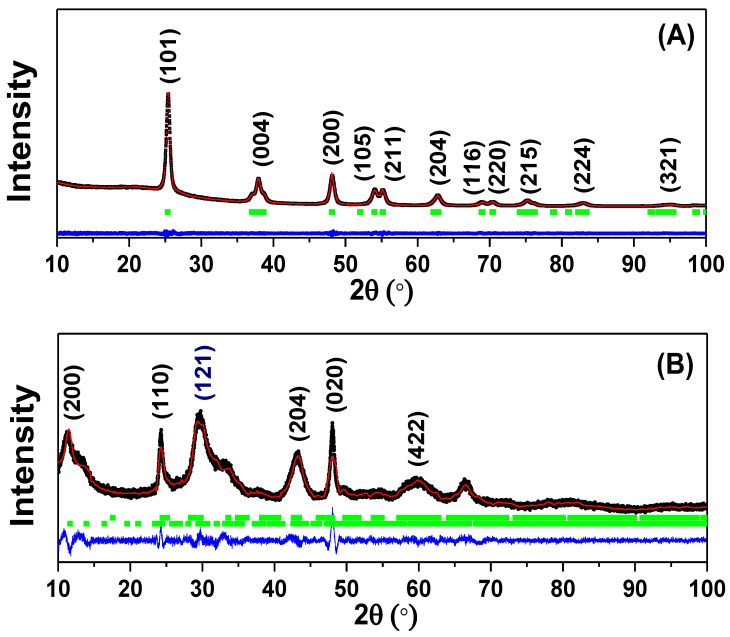
Rietveld refinement of X-ray diffractograms obtained for (**A**) TiO_2_ NPs and (**B**) TiO_2_ NWs. The black line corresponds to experimental data, red line to calculated diffractogram, and the blue line is the residual data between the experimental and calculated data. Green vertical lines indicate the Bragg’s positions and Miller indices which are given by vertical number in parentheses. Blue numbers between parentheses are assigned to brookite phase.

Refined X-ray diffractograms are presented in Figure 1 for TiO_2_ NPs (A) and TiO_2_ NWs (B). The refined X-ray diffractogram of commercial TiO_2_ NPs, Figure 1A, consists of the reported polymorph phase of TiO_2_, anatase, synthesized by sol-gel method assisted with heat treatment [24]. The corresponding Bragg’s angle positions for crystalline identified phases are given in Table S1.

In the X-ray diffractogram of Figure 1B, two crystalline contributions were observed for TiO_2_ NWs, the crystalline phase percentage of the main crystalline phase (Protonic Trititanate, 63.5%) is higher than the secondary identified crystalline phase (Brookite, 36.5%). On the other hand, as was explained in [25], the second crystalline phase is a product of the ion exchange that occurs in sodium trititanate, wherein the sodium is eliminated at a temperature of 120 °C, leaving the protonic trititanate available. Their respective Miller’s indices and Bragg’s angles were found at: (200) at 11.6°, (110) at 24°, and (020) at 48° [25]. These Bragg’s angles, according to [25], appear when the calcination temperature is lower than 350 °C. However, when the temperature is higher than 550 °C, the Bragg’s angle at 11.6° completely disappears. The disappearance of Bragg’s peak (200) is attributed to dehydration caused by the increase in temperature [26]. In this case, a Bragg’s peak with Miller index (211) appears at 43.9°. Despite the fact that the synthesis method in [26] was also hydrothermal, the two employed precursors of titanium dioxide (rutile and anatase) together with a solution of sodium hydroxide (NaOH) [26] were different and we can discard the idea of high temperature synthesis due to the lack of this (211) Miller index. Hence, the temperature for hydrothermal synthesis of the TiO_2_ NWs was less than 350 °C. Moreover, the detail indexation of brookite phase is give in Table S2.

### 3.2. µ-Raman Analysis

**Figure 2:**
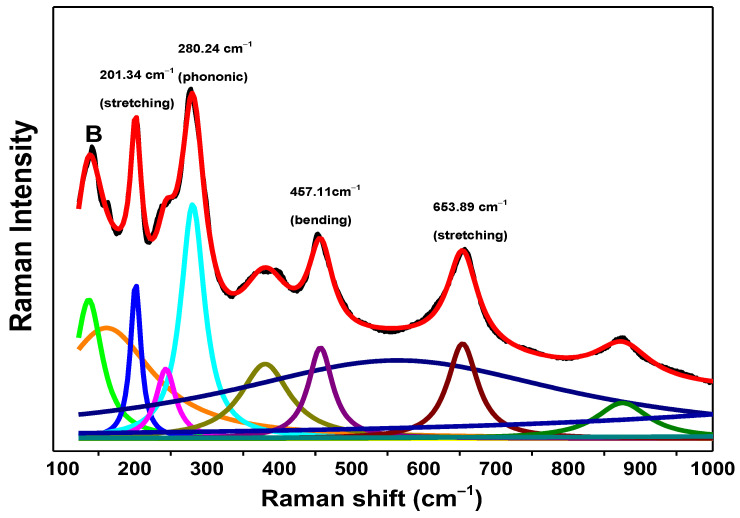
Fit to experimental µ-Raman spectrum for TiO_2_ NWs sample. The laser power over the sample was of 8.3 mW. Lorentzian subcomponents were highlighted with different colors and main Raman modes are indicated on the top of each identified active optical mode. B indicates the brookite phase.

µ-Raman spectrum for commercial TiO_2_ NPs has been previously characterized in [20]. To identify the vibrational Raman bands for the TiO_2_ NWs sample in Figure 2, a nonlinear regression was performed by means of a finite sum of Lorentzian functions. The reason the Lorentzian was taken as the profile function is because it better approximates the physical process in which inelastic light scattering intervenes [27].

Reviewing the corresponding literature, some Raman peaks or vibrational bands of the Raman curve of the TiO_2_ NWs sample were identified and are summarized in Table 1 [28,29,30].

The pure brookite phase presents between four and five characteristic vibrational Raman bands [31,32]. The strong optical signal for brookite is positioned at ~150 ${\mathrm{cm}}^{-1}$ and no other peaks were found. This is because brookite is a secondary phase and major chemical compositions are detected for the H_2_Ti_3_O_7_, which agrees with Rietveld quantitative analysis. It is worth mentioning that that the phonon mode located at 280 ${\mathrm{cm}}^{-1}$ is a characteristic vibrational band of trititanate nanotube geometries [33].

### 3.3. DLS Technique and Point of Zero Charge (p.z.c.) Determination

The effective hydrodynamic diameter (EHD) of the TiO_2_ NPs sample was determined using the DLS technique, in which the pH value of the TiO_2_ sample was varied to obtain different values of this EHD. In Figure S1, one can see the EHD for five corresponding pH values. Since TiO_2_ NWs do not have a spherical geometry, the Stokes–Einstein equation cannot, in principle, be applied, since it is valid for spherical particles only [34]. However, since they are in a fluid, they tend to agglomerate with other nanowires until they form a quasi-spherically symmetrical particle that agree with the DLS principle. The EHD measured at a pH equal to 7 of the TiO_2_ NWs was 118 nm with a base line index of 10.

**Figure 3:**
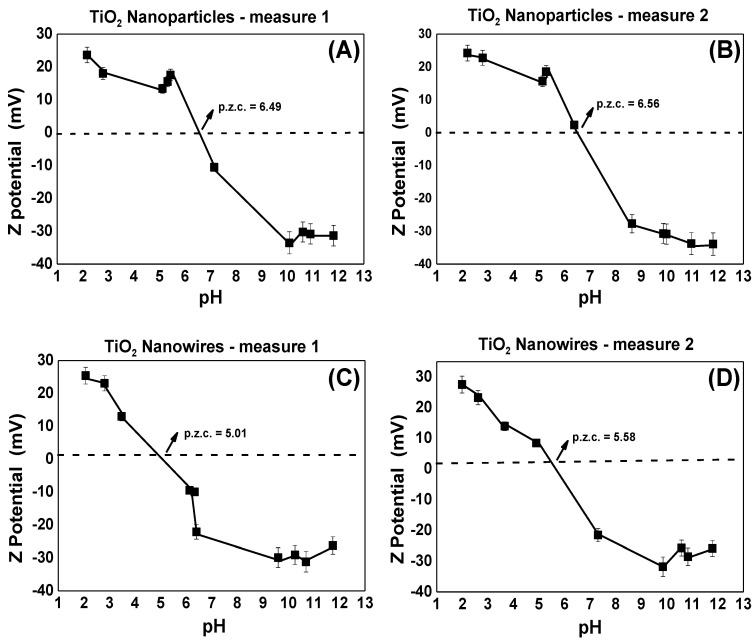
Zeta potential measurements as a function of pH for TiO_2_ samples. (**A**) First measurement for TiO_2_ NPs, (**B**) second measurement for TiO_2_ NPs, (**C**) first measurement for TiO_2_ NWs, (**D**) second measurement for TiO_2_ NWs.

The p.z.c. is the pH value corresponding to a null zeta potential value [35], or also understood as an electrical surface potential equal to zero (shear plane model). At lower pH values (below p.z.c.) it is positively charged, while at higher pH (above p.z.c.) it is negatively charged [35]. As reported by Calle et al. the p.z.c. of TiO_2_ (anatase 70% and rutile 30%) was found to be 6.5, whilst doped TiO_2_ samples have a low p.z.c. [36]. This value of 6.5 is equal to that found with our titration experiment, see Figure 3A,B, also 6.5, for anatase TiO_2_ NPs. However, for compounds that have impurities, the p.z.c. is less than 6.5. This may be the reason that the mean p.z.c. for the TiO_2_ NWs sample is 5.3, see Figure 3C,D, since it is a biphasic sample (brookite and protonic trititanate).

### 3.4. TEM Analysis

**Figure 4:**
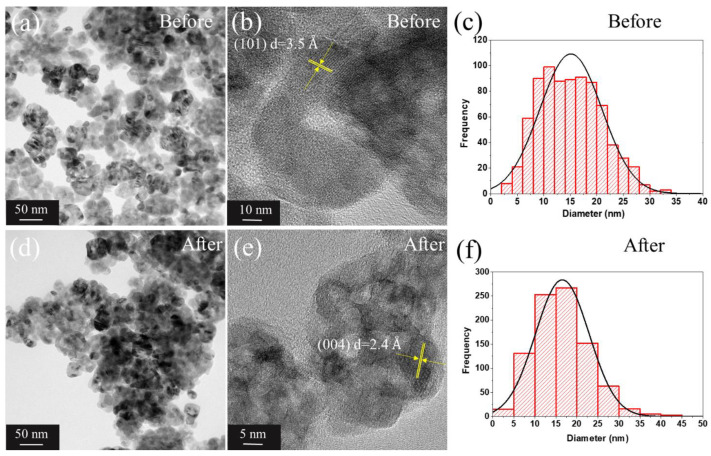
(**a**) TEM image of the TiO_2_ NPs before the ecotoxicity experiment, (**b**) 10 nm zoomed TEM image, (**c**) normal distribution of the measurements of the TiO_2_ NPs from before the ecotoxicity experiment, (**d**) TEM image of the TiO_2_ NPs after the ecotoxicity experiment, (**e**) 5 nm zoomed TEM image, and (**f**) normal distribution of the TiO_2_ NPs measurements after the ecotoxicity experiment.

**Figure 5:**
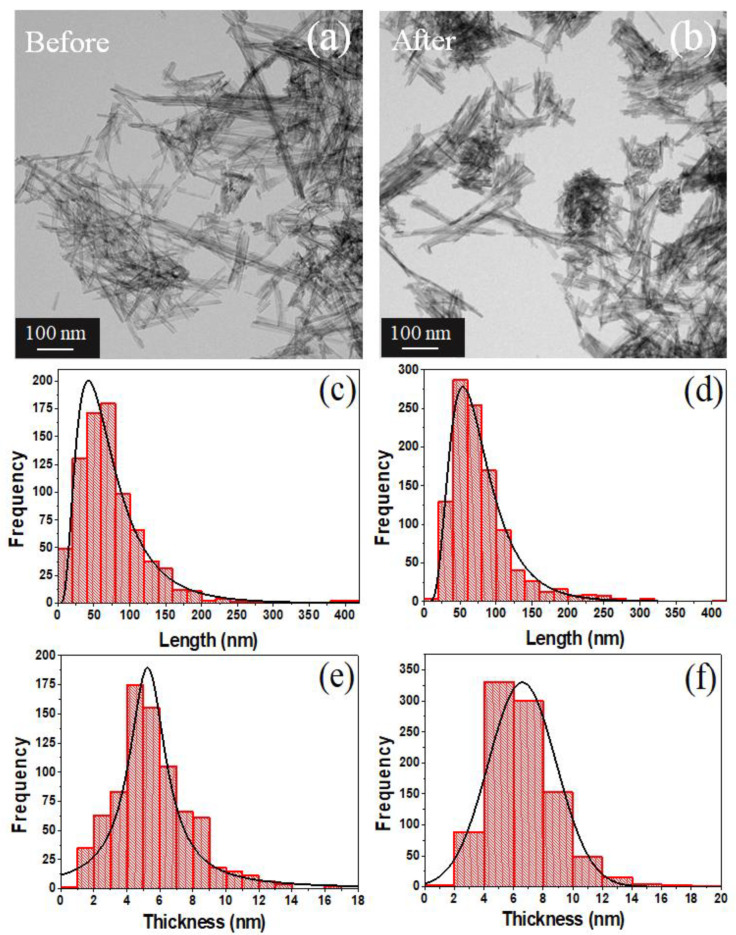
(**a**) TEM image of the TiO_2_ NWs before the ecotoxicity experiment, (**b**) TEM image of the TiO_2_ NWs after the ecotoxicity experiment. (**c**) Log-normal distribution of the TiO_2_ NWs length measurements before the experiment of ecotoxicity and (**d**) log-normal distribution of the measurements of the length of the TiO_2_ NWs after the ecotoxicity experiment. (**e**) Lorentz distribution of the measurements of the diameter of the TiO_2_ NWs before the ecotoxicity experiment and (**f**) normal distribution of the measurements of the diameter of the TiO_2_ NWs after the ecotoxicity experiment.

Figure 4a,b,d,e and Figure 5a,b show the TEM images, while Figure 4c,f and Figure 5c–f their respective particle size distribution (PSD) histograms for TiO_2_ NPs and TiO_2_ NWs, the obtained statistical parameters after fitting with a normal and log-normal distribution are listed in Table 2. This was achieved with the aim of comparing with the manufacturer’s measurements (Sigma-Aldrich) that reported 20 nm for mean particle size, in case of TiO_2_ NPs, and 10 nm (thickness)/10 µm(length) for TiO_2_ NWs. For the case of TiO_2_ NPs, the difference between before (15.1 nm) and after (16.5 nm) did not show a noticeable difference with respect to the agglomeration of TiO_2_ NPs, this was also notice in the interplanar distance of 3.5 Å for (101) and 2.4 Å for (004) planes, in agreement with Rietveld refinement, cif files in Section 2.2, and supporting information in Tables S1 and S3. However, some morphological changes to the measurements reported by the manufacturer were found in the thickness and length parameters (before exposure) for TiO_2_ NWs. The estimated thickness was less than 10 nm and the mean length value was 74.6 nm. These values remain close after performing the ecotoxicological experiments. The small value for the reported length can be related to the small temperature used for the synthesis (<350 °C), as discussed in Section 3.1. It is worth mentioning the importance of performing a rigorous characterization to correctly determine the *LC_50_* and morphological *D. magna* changes, as we will discuss in the following sections.

### 3.5. Acute Toxicity of TiO_2_ in D. magna

Over the course of 24 h, *D. magna* neonates were exposed to five different concentrations of TiO_2_ NPs and NWs. 0% of mortality was found for 75 mg L^−1^ (TiO_2_ NPs) and 800 mg L^−1^ (TiO_2_ NWs). The surviving *daphnias* were assessed for an additional 15 days in a beaker with conditions before exposure in a volume of 200 mL of *daphnia* water to determine what toxic effects they may have encountered (morphological and reproduction rate experiments).

#### 3.5.1. Lethal Concentration 24-h *LC_50_*

**Figure 6:**
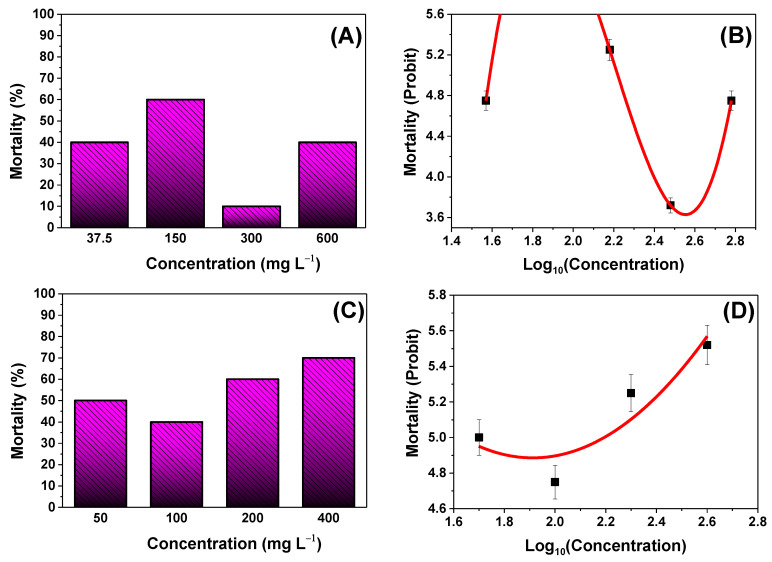
(**A**) Mortality (%) for the TiO_2_ NP sample vs. concentration and (**B**) probit of mortality vs. log10 concentration (right) fitted with a non-linear cubic function. Goodness of fit ($R^{2}$ = 1.0). (**C**) Mortality (%) for the TiO_2_ NWs sample against concentration and (**D**) mortality probit vs. log10 concentration (right) fitted with a nonlinear parabolic function. $R^{2}$ = 0.859.

As previously mentioned, 10 neonates of *D. magna* for concentration were used for 24-h *LC_50_* determination. Modified Probit analysis was used to calculate the 24-h *LC_50_* for TiO_2_ samples. Figure 6A,C show mortality (%) as a function of concentration (mg L^−1^) for both TiO_2_ samples.

In Figure 6B, a cubic regression was performed on the experimental data. The non-linear regression has a goodness of fit of 1.0 and has the following equation:

(1) $$Y=17.13\;X^{3}-113.22\;X^{2}+243.15\;X-164.2$$

In this case, substituting the value of 5.0, which is the probit value of 50% mortality, into Equation (1) gave us two points of intersection, two values (positive and negative) for the *LC_50_*. These points lie at the probit numerical values of 1.58 and 2.22. Therefore, the positive value that lies in the *LC_50_* range of various previous investigations [8,9,10,37,38] for TiO_2_ NPs was chosen. Hence, the concentration at which the *LC_50_* occurs was 166 mg $L^{-1}$.

In Figure 6D, a quadratic regression was performed on the experimental data. The non-linear regression has an R^2^ = of 0.859 and has the following equation:

(2) $$Y=1.430\;X^{2}-5.445\;X+10.062$$

The *LC_50_* for the TiO_2_ NWs sample was calculated, replacing probit value of 5.0 in Equation (2), we obtained the *LC_50_* of 157 mg $L^{-1}$.

*LC_50_* of TiO_2_ nanosystems have been shown to be dependent of structure, morphology, sample preparation method, size, specific surface area, and exposure time [8,9,10,37,38].

According to Murali et al. [9], our reported *LC_50_* values are within the range of the other compared assays of TiO_2_ NPs in *D. magna*, this range was reported to be between 118 mg $L^{-1}$ and 218.79 mg $L^{-1}$. Table 3 lists the *LC_50_* values for some TiO_2_ nanosystems found in the literature. Similarly, Johari and Ashagari have reported that the *LC_50_* was greater than 200 mg L^−1^ [10]. However, only poor references considered the importance of the EHD when comparing the *LC_50_*. The values reported by Murali et al. [9] and Gökçe et al. [37] referenced only the mean particle size, but direct exposition occurred with suspended NPs, indicating that mean hydrodynamic size needs to be determined. In our case, we used short exposure times of 24 h because of the fast response of TiO_2_ nanosystems to the adsorption of heavy metals, as, for example, in [16,39]. Hence, the 24-h *LC_50_* gave us insights into the concentration limits for quick water treatment and short exposure time for the *D. magna* individuals under the tested concentrations.

More importantly, the *LC_50_* determination was observed to depend on the solution preparation method. When the method of stock solution preparation is the same, that is, by sonication [10], it was possible to determine *LC_50_* values. However, the studied particle sizes are much larger. A notable difference is found in [8], in which the *LC_50_* could not be found by sonication, having applied concentrations of up to 500 mg L^−1^ and using a nanoparticle size greater than 100 nm. However, when the solution was prepared by filtration [8], the *LC_50_* was found to be 5.5 mg L^−1^ at a very low concentration, using the average NPs size of 30 nm. Hence, solubility of TiO_2_ NPs is an important parameter for the accurate determination of *LC_50_* values. In our case, the zeta potential values of TiO_2_ nanosystems are both ~−20 mV at pH = 7, indicating high colloidal stability.

#### 3.5.2. Morphological Analysis

Figure S2 shows the typical growth in the morphological parameters that one would expect when they are not exposed to the TiO_2_ sample, that is, the growth that *D. magna* should have naturally. Figure S3 shows the optical microscopy image of a negative control *D. magna* specimen on the first and the last day. 

**Figure 7:**
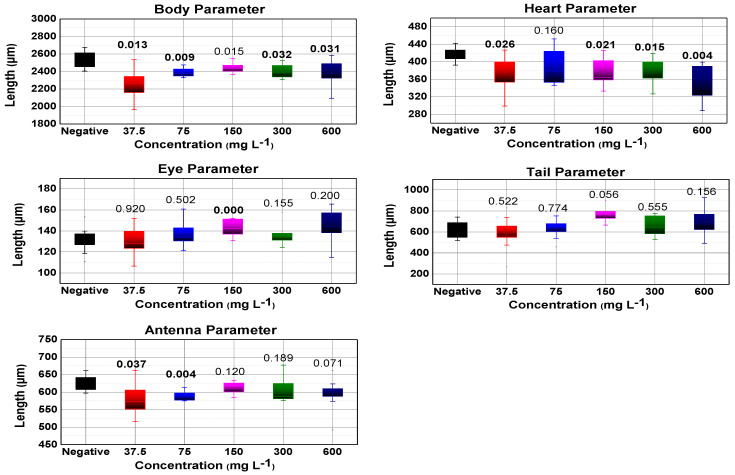
Boxplot for all morphological parameters (tail, body, heart, antenna, and eye) measured after TiO_2_ NPs exposure. Numbers above boxes are their *p*-values, with those written in bold numbers indicating significance (*p*-value < 0.05).

**Figure 8:**
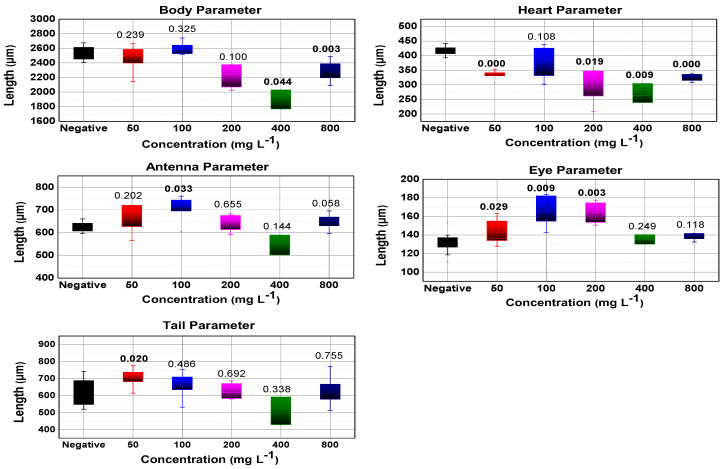
Box plot for all morphological parameters (tail, body, heart, antenna, and eye) measured after TiO_2_ NWs exposure. Numbers above boxes are their *p*-values, with those written in bold numbers indicating significance (*p*-value < 0.05).

In the boxplot presented in Figure 7 and Figure 8, the morphological parameters are illustrated, a significant change in the heart morphological parameter was evidenced for both TiO_2_ samples, this may indicate a stress level of *D. magna*, while the tail morphological parameter does not show substantial significance for both TiO_2_ samples. 

Regarding the body morphological parameter, it is shown that there is not much significance for the TiO_2_ NWs in two of the five concentrations, so there is no effect on their growth as concluded by Lee et al. [40], while for the TiO_2_ NPs there is a significance considerable in four of the five concentrations in opposition to Lee et al. [40]. In the morphological parameter antenna, no noticeable significance is observed in the five tested concentrations of both TiO_2_ nanosystems. Therefore, the erratic behavior in the swimming of *D. magna* is not confirmed to be due to this malformation. This is also corroborated by the observations of Lovern et al. [8].

**Figure 9:**
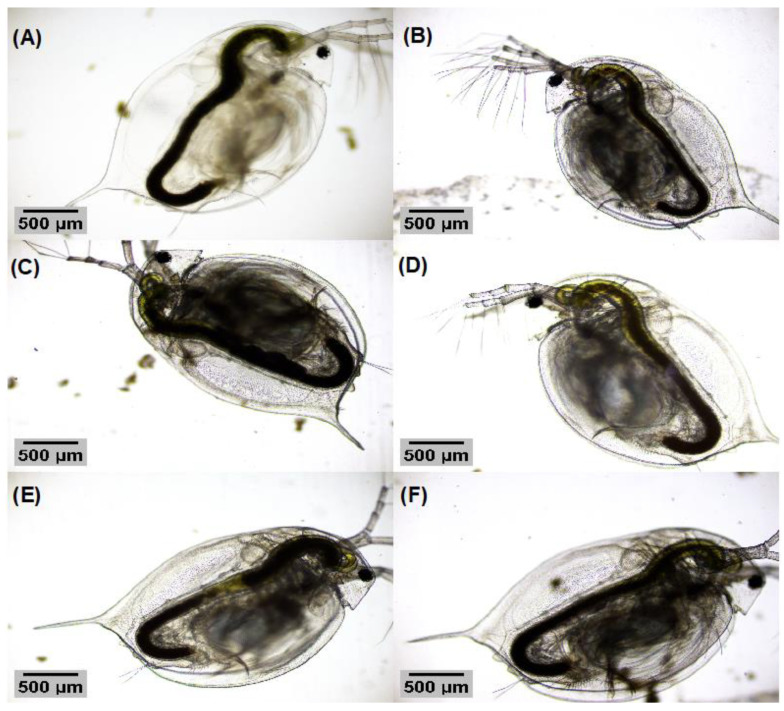
(**A**) *D. magna* individual from the negative control, (**B**) *D. magna* individual exposed to 37.5 mg $L^{-1}$ of TiO_2_ NPs, (**C**) *D. magna* individual exposed to 75 mg $L^{-1}$ of TiO_2_ NPs, (**D**) *D. magna* individual exposed to 150 mg $L^{-1}$ of TiO_2_ NPs, (**E**) *D. magna* individual exposed to 300 mg $L^{-1}$ of TiO_2_ NPs, and (**F**) *D. magna* individual exposed to 600 mg $L^{-1}$ of TiO_2_ NPs.

**Figure 10:**
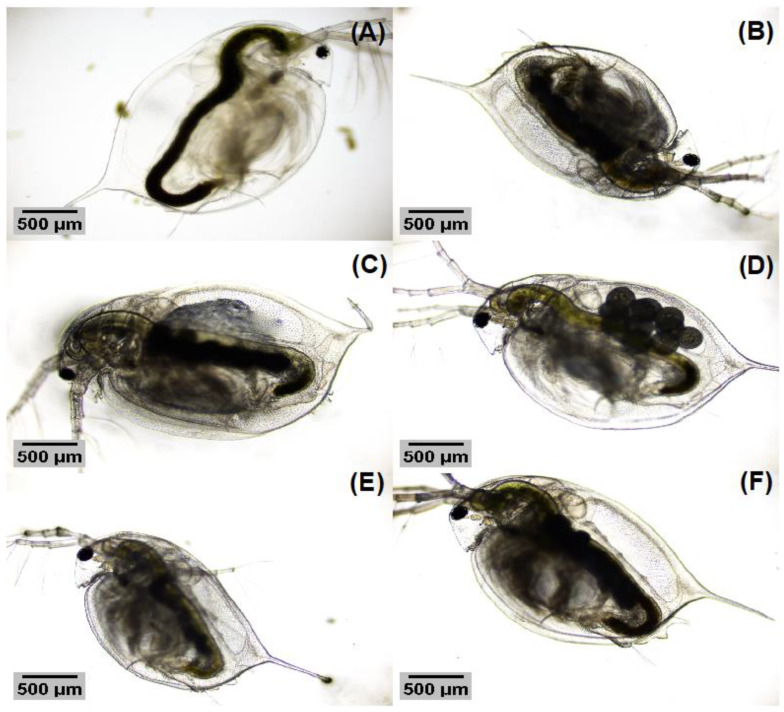
(**A**) *D. magna* individual from the negative control, (**B**) *D. magna* individual exposed to 50 mg $L^{-1}$ TiO_2_ NWs, (**C**) *D. magna* individual exposed to 100 mg $L^{-1}$ TiO_2_ NWs, (**D**) *D. magna* exposed to 200 mg $L^{-1}$ TiO_2_ NWs, (**E**) *D. magna* individual exposed to 400 mg $L^{-1}$ TiO_2_ NWs, and (**F**) *D. magna* exposed to 800 mg $L^{-1}$ TiO_2_ NWs.

Figure 9A–F and Figure 10A–F show the comparison of *D. magna* when they are exposed to both TiO_2_ samples at the tested concentrations. As can be seen, no apparent malformations were directly noticed when analyzing the optical images and no residuals of the TiO_2_ nanosystems were noticed on the body surface.

#### 3.5.3. Reproduction Rate

The reproduction rate of *D. magna* occurred in the negative control in the ninth day with 61 pups born. On day 12, however, 40 pups were born, and finally on the fifteenth day, three pups were born giving 104 individuals born (control) in parallel to the exposure experiments. Regarding the individuals of *D. magna* exposed to the TiO_2_ samples, no reproduction was observed in the period of the ecotoxicity experiment with TiO_2_ NWs. In case of TiO_2_ NPs, only two concentrations reported neonates, they were 300 mg L^−1^ (25 neonates) and 600 mg L^−1^ (20 neonates). It was also found from the morphological experiments that there was a delay in the reproduction of these individuals. Significant inhibition of growth and reproduction has been observed in chronic toxicity studies with TiO_2_ [41], while for the *D. magna* exposed to TiO_2_ there was a delay in their reproduction, this was observed due to the translucent properties of the crustaceans, which were monitored with the help of an optical microscope, and through observation of the eggs on the specie incubation chamber on the last day.

### 3.6. After Exposure Properties of TiO_2_ Nanomorphologies

Refined X-ray diffractograms for TiO_2_ samples after performing ecotoxicological experiments are given in Figure S4. In Figure S4A the characteristic Bragg’s peaks of TiO_2_ anatase are shown after the ecotoxicity experiment. These were identified using Match v3 software, confirming the crystalline phase of anatase (# 900-8214). However, some new Bragg’s peaks, not related to anatase, were observed [42]. A possible explanation for these Bragg’s peaks is the presence of sodium chloride or another minor impurity, though this cannot be elucidated with accuracy due to the predominance of TiO_2_ anatase (>90 wt. %). In Figure S4B, the refined X-ray diffractogram for TiO_2_ NWs (after the ecotoxicity experiment) permitted the identification of H_2_Ti_3_O_7_ (# 433-6946). This crystalline phase is like that shown in [43,44], wherein H_2_Ti_3_O_7_ is the product of the deionization process of Na_2_Ti_3_O_7_. The corresponding Bragg’s angle positions for identified crystalline phases are given in Tables S3 and S4.

On the other hand, the brookite phase did not appear in the phase identification using the Match v3 software, since no characteristic peak of this TiO_2_ polymorph appears in the X-ray diffractogram [42]. This may be due to the aqueous solution exposure (culture solution) with *D. magna* that causes a segregation or dilution for this secondary phase [45]. However, this phase was corroborated by analyzing the selected area electron diffraction (SAED) pattern.

**Figure 11:**
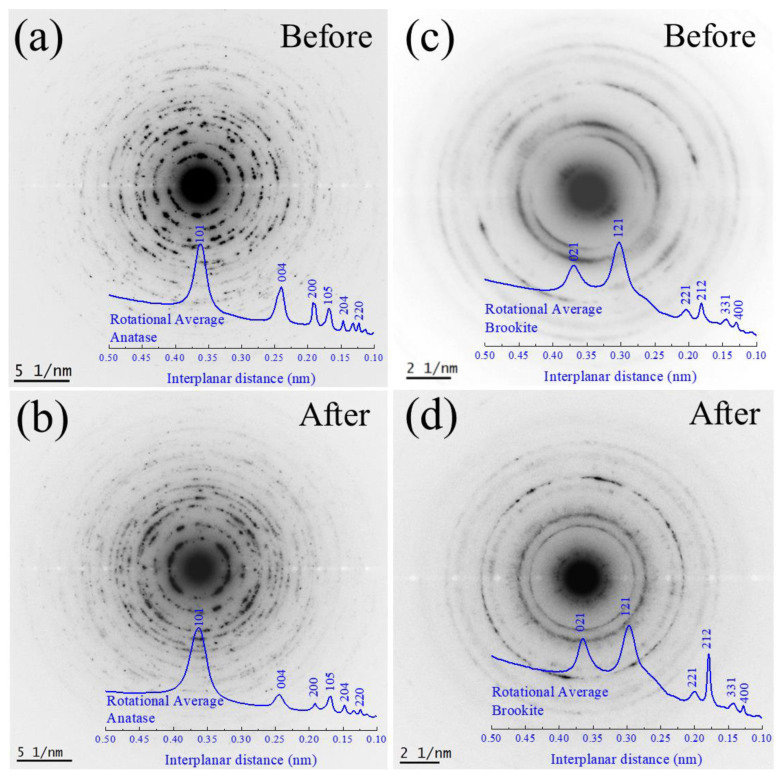
SAED pattern for before (**a**) and after (**b**) TiO_2_ NPs. SAED pattern for before (**c**) and after (**d**) TiO_2_ NWs. The inset (bottom-right) in blue indicates the indexed rotational average pattern for the identified crystalline phases before and after ecotoxicological experiments.

Figure 11a–d shows the SAED pattern for the recovered samples with their respective indexed rotational average patterns. By analyzing before and after the structural properties, it was possible to confirm the presence of the brookite phase (as a minority phase). Hence, the fingerprints indexes confirmed the presence of anatase TiO_2_ and brookite phases before and after ecotoxicological experiments.

**Figure 12:**
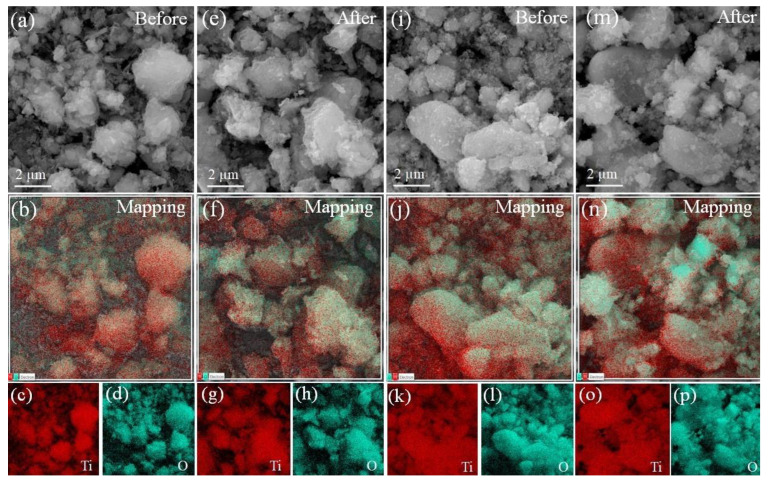
(**a**) SEM image, (**b**) EDS mapping, (**c**,**d**) elemental (Ti,O) EDS images for before TiO_2_ NPs, (**e**) SEM image, (**f**) EDS mapping, (**g**,**h**) elemental (Ti,O) EDS images for after TiO_2_ NPs, (**i**) SEM image, (**j**) EDS mapping, (**k**,**l**) elemental (Ti,O) EDS images for before TiO_2_ NWs, (**m**) SEM image, (**n**) EDS mapping, and (**o**,**p**) elemental (Ti,O) EDS images for after TiO_2_ NWs.

Figure 12a–p shows the EDS mapping images that was performed to corroborate the total weight composition for the samples and to confirm the presence of Ti and O elements homogenously distributed in the samples. Figure 12 depicts the EDS mapping images that were used to validate the overall weight composition of the samples and the existence of Ti and O elements that were evenly distributed in the samples.

Table 4 shows the quantitative weight composition of the four samples. After ecotoxicological investigations, there was a change in the weight composition of TiO_2_ NPs for Ti and O. This was mostly due to the experiment conditions and *D. magna* exposure (minority phases detected by X-ray diffraction). In contrast, no significant changes in TiO_2_ NWs composition were observed following the biological studies.

**Figure 13:**
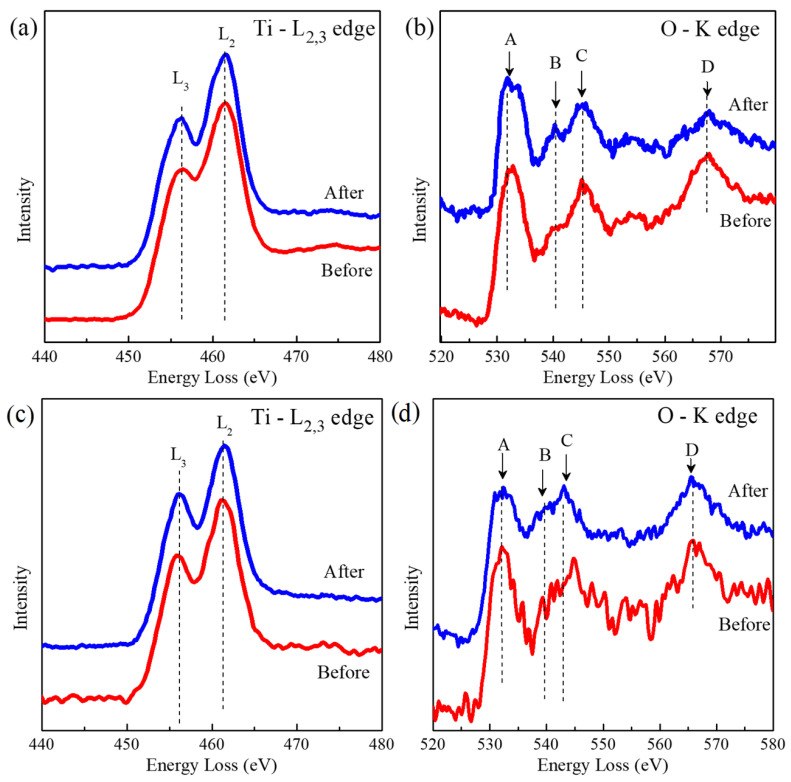
(**a**) Ti-L_2,3_ edge and (**b**) O-K edge in the EELS spectra for before and after TiO_2_ NPs. (**c**) Ti-L_2,3_ edge and (**d**) O-K edge in the EELS spectra for before and after TiO_2_ NWs. The upper letters (A, B, C, and D) indicate the characteristic energy loss positions (eV).

The O-K edge and Ti L_2,3_ edge EELS spectra for the anatase TiO_2_ NPs (before and after) are given in Figure 13a. Two peaks at 455 and 461 eV are related to L_3_ and L_2_ transitions. While the O-K edge exhibited four marked peaks given by capital letters (A–D) in Figure 13b. For both TiO_2_ NPs, four strong peaks in the O-K edge were seen at (A) 531 eV, (B) 540 eV, (C) 545 eV, and (D) 568 eV. All of these peaks were anatase TiO_2_ NPs [46,47]. There were no further peaks associated with other TiO_2_ phases before or following TiO_2_ NPs. Rutile is frequently distinguished by three extra peaks positioned after the A and B peaks that were not seen in the EELS spectra [46]. For TiO_2_ NWs in Figure 13c,d, energy peaks located at 532 eV, (B) 540 eV, (C) 543 eV, and (D) 566 eV assigned to TiO_2_ polymorphs were found, indicating that, despite the different morphologies, both systems retained their chemical structure (before and after ecotoxicological experiments).

### 3.7. Perspectives

One important subject for future research is the evaluation of how TiO_2_ NPs modifies the bioaccumulation of heavy metals in *D. magna*. Poor references are available in the literature focusing on this issue. For example, Wang et al. [48] innovatively showed that bioaccumulation of heavy metal increases to 85% in the presence of TiO_2_ NPs, and that this increase was correlated with the heavy metal’s physical properties. Hartmann et al. [49] studied how TiO_2_ NPs affected the toxicity of cadmium to aquatic organisms. While the NPs could potentially carry cadmium, their addition did not change the toxicity to the studied organisms. However, more research is needed in this direction to understand the role of nanoparticle-bound versus soluble cadmium in uptake and toxicity. Finally, experiments at molecular levels will also be needed to improve understanding and obtain a total ecotoxicological evaluation.

## 4. Conclusions

Two different commercial TiO_2_ nanomorphologies were characterized to understand their ecotoxicological properties in *D. magna*. Refined X-ray diffractograms showed that the TiO_2_ NPs sample has a monophasic anatase phase. Meanwhile, the TiO_2_ NWs biphasic sample allowed us to identify and elucidate brookite (tetragonal) and protonic trititanate (monoclinic) phases. From the quantitative analysis, 63.5 wt. % corresponded to protonic trititanate and 36.5 wt. % for brookite. Raman spectroscopy proved the presence of both phases through their optical vibrational modes. DLS allowed the calculation of the effective hydrodynamic sizes at various pH and the zeta potential values of ca. −20 mV for both TiO_2_ nanomorphologies, which are important parameters to establish the *LC_50_* and are not often studied when determining this value. The zeta potential was observed to increase in an alkaline medium for TiO_2_ samples. This means that a negative potential can favor the adsorption of divalent cations in polluted water. However, this can represent a flaw because changing the pH can affect the *D. magna* habitat; thus, future evaluations in the *LC_50_* will be needed. Therefore, the *LC_50_* values must be first determined. TEM images and statistical analysis revealed no morphological changes for TiO_2_ species (before and after ecotoxicological experiments). The ecotoxicological impact of TiO_2_ on the environmental bioindicator was slightly higher for TiO_2_ NWs than TiO_2_ NPs, despite their close *LC_50_* values of 157 and 166 mg L^−1^, the mortality in TiO_2_ NWs was high and equivalent to 50% for the three of five concentrations but mortality in TiO_2_ NPs was only above 50% in one of the five tested concentrations. These nanomaterials also caused a delay in *D. magna* reproduction, because, in favorable conditions (negative control), the neonates began to be born on day nine, having 104 *D. magna* individuals counted for control (after 16 days). However, those *daphnids* exposed to TiO_2_ NWs had no neonates at the end of the experiments under the tested concentrations. On the contrary, neonates were only born in the last day of the experiments for 300 mg L^−1^ (25 neonates) and 600 mg L^−1^ (20 neonates) TiO_2_ NPs. This may represent an important disadvantage that must be carefully evaluated in future works and damage comparison with studies at molecular levels will be further necessary. In terms of morphological metrics, it was found that the heart size for *D. magna* varies significantly when exposed to TiO_2_ samples at four of the five tested concentrations. It was also observed that there was no change in *D. magna* swimming because there was no overall significant change in the antenna parameter after TiO_2_ nanomorphologies exposure, which is the morphological feature that permits *D. magna’s* distinctive jump. As a result of this research, we can infer that the toxic effects of TiO_2_ NWs are more severe than those of TiO_2_ NPs. Finally, we went beyond the ecotoxicological experiments by analyzing the recovering samples by X-ray diffraction, SAED, EDS mapping, and EELS, revealing that the structure and morphological properties of TiO_2_ nanomorphologies are kept after the biological experiments. This represents a potential improvement, since they can be recycled and stored for future applications, such as water remediation, below the determined *LC_50_* values.

## Figures and Tables

**Table 1:** Vibrational Raman bands of the TiO_2_ NWs sample.

Crystalline Phase	Raman Shift (cm^−1^)	Vibrational Assignment
H_2_Ti_3_O_7_	201.34	Vibration mode (stretching)
H_2_Ti_3_O_7_	280.24	Phonic mode
H_2_Ti_3_O_7_	457.11	Vibration mode (Bending)
H_2_Ti_3_O_7_	653.89	Vibration mode (stretching)

**Table 2:** TEM parameters obtained for the TiO_2_ samples after fitting the data with normal and log-normal distribution. <D> is the mean particle size.

Samples	<D> (nm)	Standard Deviation	Polydispersity
Before TiO_2_ NPs	15.1	5.9	0.39
After TiO_2_ NPs	16.5	6.4	0.39
Before TiO_2_ NWs	5.6 (thickness)	2.3	0.42
	74.6 (length)	48.3	0.65
After TiO_2_ NWs	6.6 (thickness)	2.3	0.35
	79.2 (thickness)	46.4	0.59

**Table 3:** *LC_50_* values for TiO_2_ nanosystems exposed to *D. magna*. (n.d. = Not determined).

Nanosystem	Mean Particle Size in Aqueous Media	NPs Source	Exposition Time (h)	*LC_50_* (mg L^−1^)	Reference
TiO_2_ NPs	n.d.	Synthesized in the lab	48	>100	[8]
TiO_2_ NPs	15–500 nm	Commercial	48	>200	[10]
TiO_2_ NPs	n.d.	Synthesized in the lab	96	1.8	[37]
TiO_2_ NPs	0.5–70 µm	Commercial	48	>100	[38]
TiO_2_ NPs	130 nm	Commercial	24	166	This work
TiO_2_ NWs	118 nm	Commercial	24	157	This work

**Table 4:** Quantitative weight composition obtained from EDS mapping.

Sample	Ti (%wt)	O (%wt)
Before TiO_2_ NPs	59.1 (1.5)	40.9 (1.5)
After TiO_2_ NPs	72.3 (0.2)	27.7 (0.2)
Before TiO_2_ NWs	55.7 (1.5)	44.3 (1.5)
After TiO_2_ NWs	50.3 (0.4)	49.7 (0.4)

## Data Availability

The original data related to this research can be asked for any time to the corresponding author’s email: juan.ramos5@unmsm.edu.pe.

## Supplementary Materials

The following supporting information can be downloaded at: https://www.mdpi.com/article/10.3390/nano13050927/s1. Figure S1: Graph of EHD vs. pH for TiO_2_ NPs (anatase), each point is the result of the measurement with the highest baseline index; Figure S2: Box plot for all morphological parameters (tail, body, heart, antenna and eye) for the negative control for both the first day and the last day; Figure S3: (A) *D. magna* neonate and (B) 16-day-old *D. magna*; Figure S4: Rietveld refinement of the diffractogram obtained for after the ecotoxicity experiment, (A) TiO_2_ NPs and (B) TiO_2_ NWs; Table S1: crystalline phase, Miller indices, and the Bragg angle for the TiO_2_ NPs; Table S2: crystalline phase, Miller indices, and the Bragg angle for the TiO_2_ NWs sample; Table S3: crystalline phase, Miller indices, and Bragg angle for the TiO_2_ NPs after ecotoxicity experiment; and Table S4: crystalline phase, Miller indices, and Bragg angle for the TiO_2_ NWs after ecotoxicity experiment.
