# Supplementary material for: Ecotoxicological Properties of Titanium Dioxide Nanomorphologies in Daphnia magna
Source: Nanomaterials (Basel). 2023 Mar 3;13(5):927. doi: 10.3390/nano13050927 (PMC10005163; doi:10.3390/nano13050927)
Supplement: Supplementary file 1 [file nanomaterials-13-00927-s001.zip › nanomaterials-2237619-supplementary.pdf]

nanomaterials

Article

Supplementary material for Ecotoxicological properties of titanium dioxide nanomorphologies in *Daphnia magna*

Freddy Mendoza-Villa[1], Noemi-Raquel Checca-Huaman[2], Juan A. Ramos-Guivar[1,*]

[1] Grupo de Investigación de Nanotecnología Aplicada para Biorremediación Ambiental, Energía, Biomedicina y Agricultura (NANOTECH), Facultad de Ciencias Físicas, Universidad Nacional Mayor de San Marcos, Av. Venezuela Cdra 34 S/N, Ciudad Universitaria, Lima 15081, Perú

[2] Centro Brasileiro de Pesquisas Físicas (CBPF), R. Xavier Sigaud, 150, Urca, Rio de Janeiro, 22290-180, Brazil

* Correspondence: juan.ramos5@unmsm.edu.pe (J.A.R.-G.)

Supplementary Figures

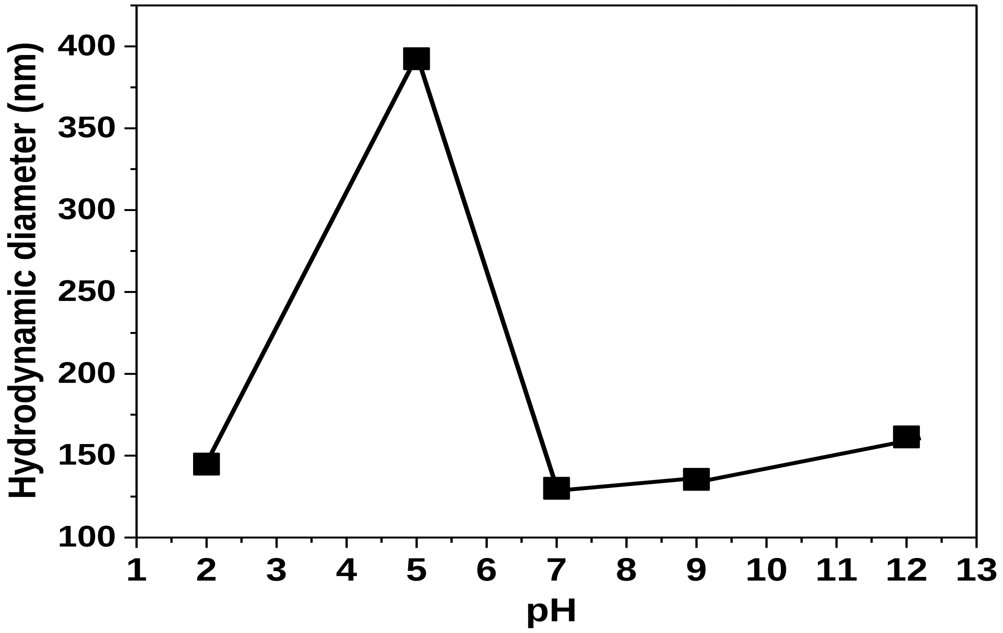

Figure S1. Graph of EHD vs pH for TiO_2 NPs (anatase). Each point is the result of the measurement with the highest baseline index.

nanomaterials

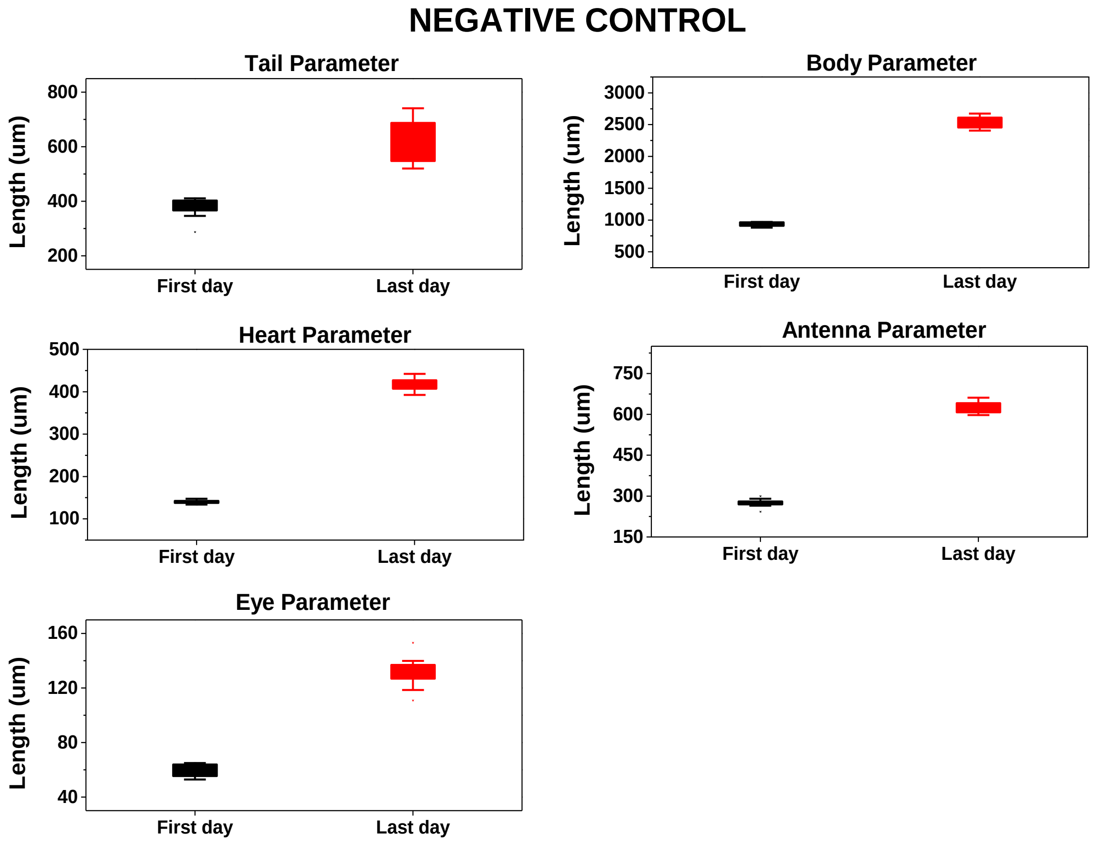

Figure S2. Box plot for all morphological parameters (Tail, Body, Heart, Antenna and Eye) for the negative control for both the first day and the last day.

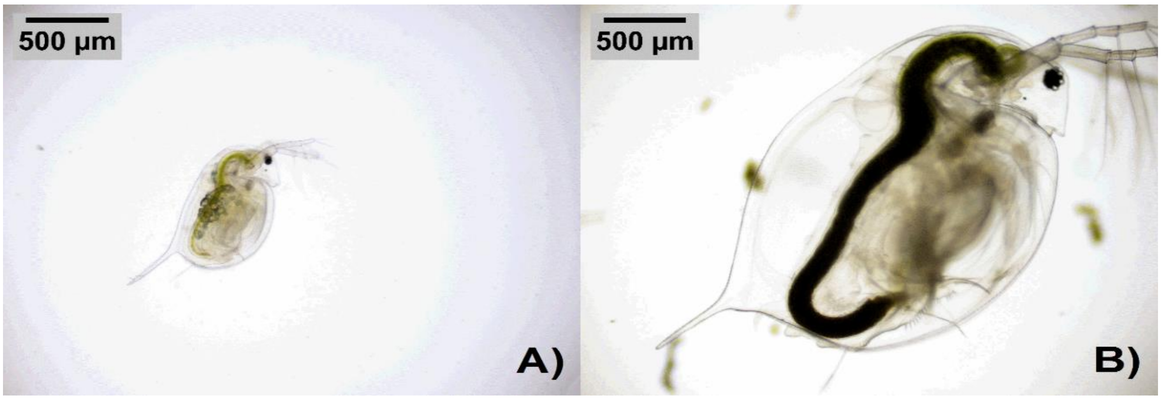

Figure S3. (A) *D. magna* neonate and (B) 16-day-old *D. magna*.

nanomaterials

MDPI

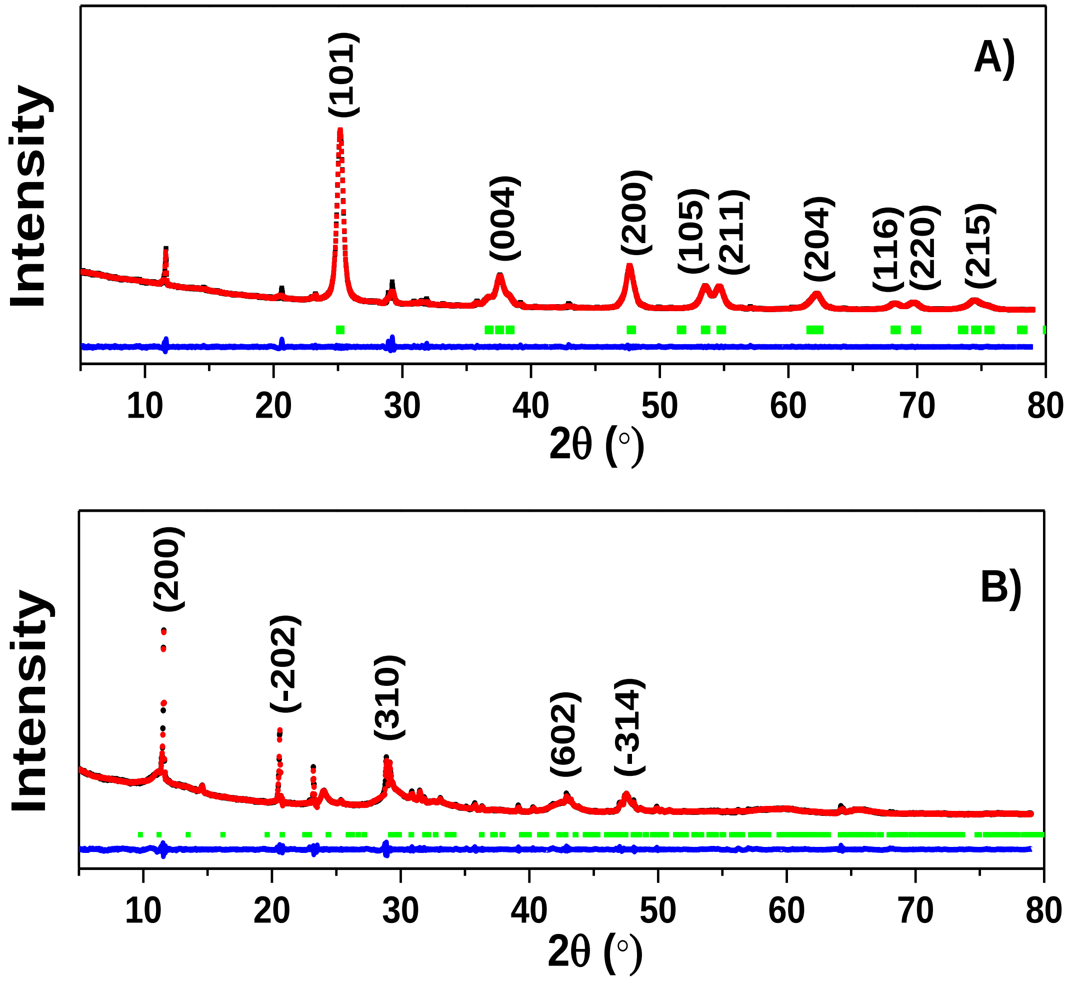

Figure S4. Rietveld refinement of the diffractogram obtained for the after the ecotoxicity experiment (A) TiO_2 NPs and (B) TiO_2 NWs.

nanomaterials

MDPI

Supplementary Tables

Table S1: Crystalline phase, Miller indices, and the Bragg angle for the TiO_2 NPs.

Crystalline phase	Miller indices	Bragg angle
anatase TiO_2	(101)	25.3 °
anatase TiO_2	(004)	37.8 °
anatase TiO_2	(200)	48.0 °
anatase TiO_2	(105)	53.9 °
anatase TiO_2	(211)	55.0 °
anatase TiO_2	(204)	62.7 °
anatase TiO_2	(116)	68.8 °
anatase TiO_2	(220)	70.3 °
anatase TiO_2	(215)	75.1 °
anatase TiO_2	(224)	82.7 °
anatase TiO_2	(321)	95.2 °

Table S2: Crystalline phase, Miller indices, and the Bragg angle for the TiO_2 NWs sample.

The crystalline phase	Miller indices	Bragg's angle
Protonic Trititanate $H_2O_7Ti_3$	(200)	11.6 °
Protonic Trititanate $H_2O_7Ti_3$	(110)	24.2 °
Brookite TiO_2	(121)	29.7 °
Protonic Trititanate $H_2O_7Ti_3$	(204)	43.4 °
Protonic Trititanate $H_2O_7Ti_3$	(020)	48.0 °
Protonic Trititanate $H_2O_7Ti_3$	(422)	59.5 °

Table S3: Crystalline phase, Miller indices, and Bragg angle for the TiO_2 NPs after ecotoxicity experiment.

Crystalline phase	Miller indices	Bragg's angle
anatase TiO_2	(101)	25.3 °
anatase TiO_2	(004)	37.8 °
anatase TiO_2	(200)	48.0 °
anatase TiO_2	(105)	53.9 °
anatase TiO_2	(211)	55.0 °
anatase TiO_2	(204)	62.7 °
anatase TiO_2	(116)	68.8 °
anatase TiO_2	(220)	70.3 °
anatase TiO_2	(215)	75.1 °

Table S4: Crystalline phase, Miller indices, and Bragg angle for the TiO_2 NWs after ecotoxicity experiment.

Crystalline phase	Miller indices	Bragg angle
Protonic Trititanate $H_2O_7Ti_3$	(200)	11.6 °
Protonic Trititanate $H_2O_7Ti_3$	(-202)	20.6 °
Protonic Trititanate $H_2O_7Ti_3$	(310)	29.2 °
Protonic Trititanate $H_2O_7Ti_3$	(602)	42.7 °
Protonic Trititanate $H_2O_7Ti_3$	(-314)	47.3 °
